# Exploring Acne Treatments: From Pathophysiological Mechanisms to Emerging Therapies

**DOI:** 10.3390/ijms25105302

**Published:** 2024-05-13

**Authors:** Hyun Jee Kim, Yeong Ho Kim

**Affiliations:** 1 Department of Dermatology, International St. Mary’s Hospital, College of Medicine, Catholic Kwandong University, Incheon 22711, Republic of Korea; hyunjee0921@hanmail.net; 2 Department of Dermatology, Seoul St. Mary’s Hospital, College of Medicine, The Catholic University of Korea, Seoul 06591, Republic of Korea

**Keywords:** acne genesis, therapeutics, microbiome

## Abstract

Acne vulgaris is a common dermatological condition that can present across different ages but predominantly affects adolescents and young adults. Characterized by various lesion types, the pathogenesis of acne is complex, involving genetic, hormonal, microbial, and inflammatory factors. This review comprehensively addresses current and emerging acne management strategies, emphasizing both topical and systemic treatments, procedural therapies, and dietary modifications. Key topical agents include retinoids, benzoyl peroxide, antibiotics, and other specialized compounds. Systemic options like antibiotics, hormonal therapies, and retinoids offer significant therapeutic benefits, particularly for moderate to severe cases. Procedural treatments such as laser devices, photodynamic therapy, chemical peels, and intralesional injections present viable alternatives for reducing acne symptoms and scarring. Emerging therapies focus on novel biologics, bacteriophages, probiotics, and peptides, providing promising future options. This review underscores the importance of personalized approaches to treatment due to the multifaceted nature of acne, highlighting the potential of innovative therapies for improving patient outcomes.

## 1. Introduction

Acne, a prevalent dermatological condition, affects individuals across all ages, though it predominantly occurs in adolescents and young adults. Commonly, lesions appear on the face, neck, upper back, and chest. Acne manifests in several forms, including neonatal and infantile acne, occupational acne, acne vulgaris, acne conglobata, acne fulminans, acne mechanica, acne excoriee (picker’s acne), chloracne, and acne medicamentosa. Of these, acne vulgaris is the most common, comprising 99% of cases [1,2]. It is characterized by distinct lesion types, categorized as non-inflammatory and inflammatory. Non-inflammatory lesions include both open comedones (blackheads) and closed comedones (whiteheads). Inflammatory lesions include papules, pustules, nodules, and cysts [3].

The pathogenesis of acne is multifactorial, involving sebaceous gland hyperactivity, follicular hyperkeratinization, microbial colonization (particularly by *Cutibacterium acnes*), and a consequent inflammatory response [4,5,6]. The interplay of these factors, influenced by genetic, hormonal, and environmental variables, underpins the complexity of acne management strategies.

Current treatment modalities for acne are diverse, reflecting the multifaceted nature of the condition. Treatments range from topical agents, such as retinoids and benzoyl peroxide, to systemic therapies, including antibiotics and hormonal agents. The choice of treatment is dictated by the acne type, with milder forms often managed with topical agents and more severe forms requiring systemic treatment. Recent advances have also explored the roles of diet, the gut microbiome, and novel pharmacological targets in managing acne, suggesting a move toward more personalized and effective treatment protocols. This article aims to elucidate the pathophysiological mechanisms of acne and to explore the array of conventional and emerging treatment strategies. This comprehensive review highlights the complexity of acne management and underscores the importance of personalized therapeutic approaches in contemporary dermatological practice.

## 2. Pathophysiology of Acne

**Figure 1:**
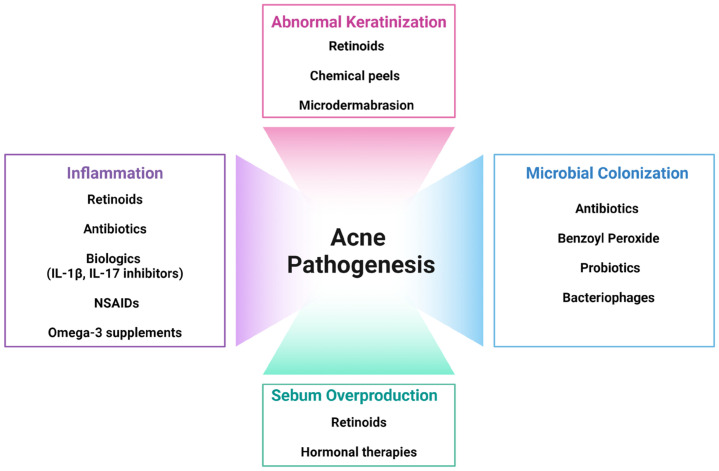
Acne pathogenesis and therapeutic targets.

The pathophysiology of acne is complex, making it a multifaceted condition that varies significantly across individuals (Figure 1).

### 2.1. Genetic and Environmental Factors

#### 2.1.1. Genetic Factors

Genetics plays a crucial role in acne development, with studies indicating a higher prevalence of acne in individuals with a family history of the condition [7,8,9,10,11,12,13,14,15,16,17,18,19,20]. Genes influence several aspects of acne pathogenesis, including sebum production, keratinization processes, and the immune system’s response to *C. acnes* [21,22,23,24,25,26]. For instance, variations in genes related to the androgen receptor can lead to increased sebum production, a key factor in acne development [23]. Similarly, genetic factors can influence the body’s inflammatory response, contributing to the formation and severity of acne lesions [21,27].

#### 2.1.2. Environmental Factors

Environmental factors are equally influential in the pathogenesis of acne. These include diet, climate, pollution, and lifestyle. Dietary habits, particularly the consumption of high-glycemic-index foods and dairy products, have been linked to acne severity, possibly due to their role in insulin-like growth factor 1 (IGF-1) signaling, which can exacerbate sebum production and inflammation [28,29,30,31,32,33,34,35,36,37,38,39,40,41,42,43]. Climate factors, such as humidity and temperature, can affect skin hydration and sebum production, while air pollutants such as NO_2_ and sulfur dioxide can increase the oxidative stress on the skin, contributing to acne flares [44,45]. Increased humidity cause epidermal keratinocyte swelling, leading to acute hair follicle occlusion, which is a favorable environment for *C. acnes* [44]. Increased sebum production following an increase in temperature also promotes the growth of lipophilic organisms such as *C. acnes* [46]. Additionally, stress has been recognized to exacerbate acne through hormonal fluctuations that increase sebum production [47].

### 2.2. Hormonal Influences

The pathophysiology of acne is profoundly influenced by hormonal factors, particularly those that alter sebaceous gland function. Androgens are pivotal in stimulating sebocyte proliferation, increasing intracellular lipid droplets, and inducing hyperkeratinization of the follicular infrainfundibulum, leading to increased sebum production and acne severity [48]. Hyperseborrhea and dysseborrhea are primary pathological conditions that have been identified, with the former indicating excessive sebum production and the latter changes in sebum composition, both of which contribute to *C. acnes* proliferation, inflammation, and comedogenesis [49].

Androgens stimulate lipid synthesis and sebocyte differentiation by enhancing the phosphorylation of mammalian target of rapamycin (mTOR), a central player in cellular metabolism, and by activating sterol regulatory-element-binding protein-1 (SREBP-1) via mTORC1, thus promoting lipogenesis [50,51]. Additionally, androgens downregulate the Wnt/β-catenin pathway, upregulating genes like c-MYC and enhancing sebocyte differentiation [52,53,54].

IGF-1 is another crucial hormonal driver, influencing acne by downregulating the nuclear transcription factor forkhead box protein O1 (FoxO1), which, in turn, increases lipogenesis and androgen receptor transduction [5,55,56]. IGF-1 signaling, critical in acne development, regulates the PI3K/Akt cascade and FoxO1 activity, impacting lipogenesis and androgen receptor (AR) transactivation and influencing lipid synthesis and sebaceous secretion [57,58,59,60,61,62,63,64,65].

Neuropeptides, including corticotropin-releasing hormone (CRH), substance P (SP), and the pro-opiomelanocortin (POMC) system, also play a significant role by modulating sebocyte activity and inflammatory responses [48,66,67]. CRH, for instance, is highly expressed in acne-prone skin, supporting a mechanism for stress-induced acne, and it interacts with androgenetic signaling pathways involved in acne pathogenesis [48].

In addition to local hormonal effects on sebaceous glands, systemic hormonal disorders play a crucial role in triggering adult acne. The onset of adult acne often necessitates an investigation into potential underlying endocrine disorders, marked by symptoms of virilization and hormonal imbalances. Clinical signs such as irregular menstrual cycles, clitoral hypertrophy, hirsutism, female-pattern hair loss, and sudden-onset or resistant acne may indicate systemic hyperandrogenism [68]. Conditions like obesity, infertility, metabolic syndrome, and hypothyroidism also merit attention for their association with hormonal disorders. Diagnostic efforts should extend to adrenal hyperplasia, virilizing tumors, and polycystic ovary syndrome (PCOS) in patients with abnormal laboratory findings [69]. Hormonal evaluations, recommended during the early follicular phase of the menstrual cycle, should encompass tests for free and total testosterone, DHEA-S, androstenedione, sex-hormone-binding globulin (SHBG), prolactin, and cortisol [68]. Findings of elevated anti-Müllerian hormone or DHEAS and low SHBG levels may indicate peripheral hyperandrogenism, underscoring the need for a comprehensive hormonal assessment in adult female acne management [68].

### 2.3. Microbiome

The skin microbiome, including *C. acnes*, coagulase-negative *Staphylococcus*, and fungal species, plays a role in maintaining skin health by balancing homeostatic relationships [70]. While the proliferation of *C. acnes* has been associated with acne, it is the diversity and specific phylotypes of *C. acnes* that are more directly implicated in acne development [71]. *C. acnes* is distinguished by unique genomic elements linked to acne pathogenesis [72]. Different strains of *C. acnes* can induce varying immune responses; acne-associated strains trigger pro-inflammatory cytokines like IFNγ and IL-17, whereas health-associated strains promote the production of anti-inflammatory IL-10 [70].

Microbial dysbiosis, particularly involving *C. acnes* and *Staphylococcus epidermidis*, contributes to acne by influencing innate immunity pathways such as PPAR and IFN signaling and TNF, IL, TLR, and MMP activation, leading to immune dysregulation, epithelial dysfunction, and overgrowth of pathogenic microbes [70,73]. *C. acnes* plays a pivotal role in acne pathogenesis by interacting with innate immunity through the release of extracellular enzymes and reactive oxygen species and the activation of TLRs, alongside influencing sebum production, keratin, filaggrin, and IGF-1 levels. This leads to inflammation and hyperkeratosis, which maintains inflammation through acquired cell-mediated responses via Th1 cells [74].

Increasing evidence also supports an interplay between the skin and gut microbiomes, enriching our understanding of acne pathogenesis beyond the skin’s surface. The intricate ecosystem within the human gut, encompassing a diverse array of bacteria, fungi, viruses, and protozoa, plays a critical role in immune modulation, nutrient synthesis, and defense against pathogens [4,75,76,77]. Dominated primarily by the bacterial phyla *Firmicutes* and *Bacteroidetes*, the gut microbiome varies significantly among individuals due to diet, antibiotics, and lifestyle influences [75,78]. This variation is implicated in the development of acne, as alterations in the gut microbiota, known as gut dysbiosis, have been observed in acne patients, characterized by reduced diversity and shifts in the balance of *Bacteroidetes* to *Firmicutes*, reflecting dietary patterns associated with a Western diet [73,79,80].

This connection between the gut and skin extends beyond mere coexistence, suggesting direct influences whereby gut microbes and their metabolic products can impact the skin microbiome via the bloodstream [76]. Such microbial interactions and the resultant modulation of cell expansion and metabolism, particularly through the mTOR signaling pathway, underscore a deeper link between gut dysbiosis and acne inflammation [51,76,81]. Furthermore, the role of microbial metabolites in systemic immune regulation is highlighted by the actions of specific gut bacteria, such as *Bacteroides fragilis*, which produce anti-inflammatory compounds, contrasting with others that might enhance inflammation, underscoring the complexity of the gut–skin axis in acne pathogenesis [76,77]. Emotional stress has been shown to alter the gut microbiome, leading to systemic and skin inflammation, further exacerbating acne [76,77]. Furthermore, gut dysbiosis can influence acne through changes in sebum composition and the promotion of pathogenic *C. acnes* strains, highlighting the role of the gut microbiome in acne pathogenesis [82].

### 2.4. Inflammation

The pathogenesis of acne is characterized by complex interactions between the innate and adaptive immune systems, where *C. acnes* plays a pivotal role. *C. acnes* triggers the activation of the Toll-like receptors TLR-2 and TLR-4 on sebocytes and keratinocytes, initiating the release of pro-inflammatory cytokines such as IL-6, IL-8, and IL-12 from monocytes, markers that are notably elevated in acne lesions [83,84]. Beyond Toll-like receptor activation, *C. acnes* also stimulates the Nod-like receptor 3 (NLRP3) inflammasome in monocytic cells, promoting the increased secretion of pro-inflammatory IL-1β [85].

The role of adaptive immunity is also critical in acne. Early acne lesions are characterized by a significant presence of CD4+ T-helper cells. The presence of CD4(+) lymphocytes around follicles in early lesions indicates that inflammation precedes hyperkeratinization [86]. This early inflammatory phase is marked by macrophages and CD3(+) and CD4(+) cells, along with activated vascular adhesion molecules like E-selectin and integrin, emphasizing inflammation’s primacy role in acne development [87]. *C. acnes* not only drives the proliferation of T-cells but also leads to the accumulation of *C. acnes*-specific T-cells [88]. These cells are induced to release IFN-γ and IL-17A, fostering a combined Th17 and Th1/Th17 host response that contributes to the inflammatory landscape of acne [85,88].

Moreover, *C. acnes*’s activation of activator protein 1 (AP1) and nuclear factor (NF)-κB leads to the production of matrix metalloproteinases (MMPs), TNFα, IL-1β, and IL-8 [89]. Elevated IL-17, IL-23, and TNFα levels in acne lesions, coupled with TLR2(+) macrophages and IL-17 cells, reveal a complex network of inflammatory pathways that exacerbate acne severity [85,90]. In the latter stages, the dominance of memory T cells, plasma cells, and B cells in atrophic scars indicates sustained immune responses [91,92]. *C. acnes*’s enzymatic activity, including lipase, hyaluronidase, and protease activities, potentially contributes to follicular wall breakdown, leading to extensive inflammation, foreign body granulomas, and scarring [93].

Additionally, IGF-1 elevates pro-inflammatory cytokine levels in primary human sebocytes, an effect attenuated by NF-κB inhibitors [94,95]. This suggests IGF-1’s significant role in initiating inflammation within the pilosebaceous unit. Androgens, by raising serum IGF-1 levels, enhance the recruitment of inflammatory cells and cytokines and MMP release [96,97]. MMPs disrupt the follicular membrane, exacerbating inflammation [98]. Furthermore, neutrophils mediate inflammation by increasing hydrogen peroxide generation, underscoring the intricate interplay of immune cells in acne pathogenesis [99].

## 3. Current Treatment Strategies

Current treatment strategies for acne focus on addressing the various factors contributing to its pathogenesis, including abnormal keratinization, increased sebum production, microbial colonization, and inflammation.

### 3.1. Topical Treatments 

**Figure 2:**
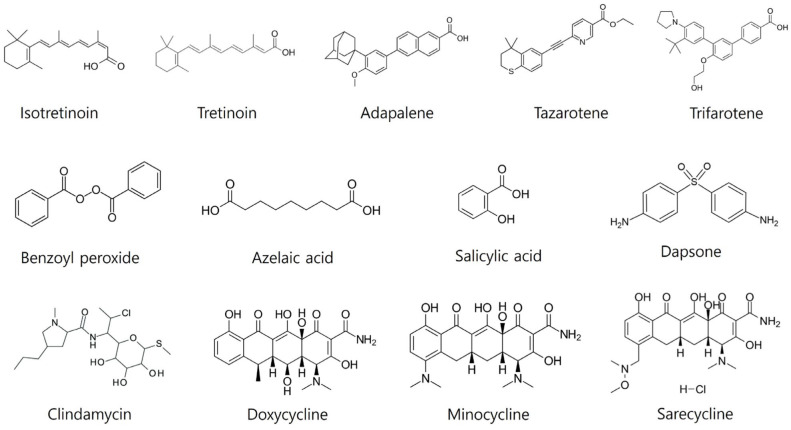
Chemical structures of the drugs used in acne therapy.

Topical treatments remain a cornerstone of acne management due to their direct action on the skin, minimal systemic side effects, and ease of use. This section reviews the efficacy and mechanisms of action of key topical agents, including retinoids, benzoyl peroxide, antibiotics, and other topical agents (Table 1, Figure 2).

#### 3.1.1. Topical Retinoids

Retinoids exert their effects through their binding to retinoic acid receptors (RARs) in the cell nucleus [100]. There are three RAR subtypes: RAR-α, RAR-β, and RAR-γ; of these, RAR-γ is the most highly expressed in human skin [101]. Retinoids are categorized into four generations based on their molecular structures and specific receptor affinities, with the latest generation progressively reducing toxicity while enhancing efficacy [102,103]. Additionally, topical retinoids are classified into six classes: tretinoin (all-trans retinoic acid), adapalene, tazarotene, trifarotene, alitretinoin, and bexarotene [102]. Among these, the United States of America Food and Drug Administration (FDA) has approved four topical retinoids (tretinoin, adapalene, tazarotene, trifarotene) for acne treatment. Topical retinoids are a cornerstone of acne therapy due to their comedolytic and anti-inflammatory effects. These vitamin A derivatives bind to retinoic acid receptors in skin cells, influencing gene transcription to increase skin cell turnover and reduce oil production [104,105]. Topical therapies offer the advantage of direct application to the affected area, minimizing potential systemic side effects and increasing exposure within the pilosebaceous follicular unit [3].

- First-Generation Retinoids

Tretinoin became the first topical retinoid to be approved for the treatment of acne by the FDA in 1971 [101]. Tretinoin has a high affinity for all three retinoic acid receptor subtypes, but its binding to RAR-γ is key to its effects [101]. Tretinoin comes in various strengths and formulations (gel, cream, microsphere, lotion) [106]. It functions to normalize the epithelial layer, prevent pilosebaceous unit occlusion, and reduce sebum production [107]. While effective, it can increase sun sensitivity, and potential side effects include redness, dryness, itching, and irritation [108,109].

b. Second-Generation Retinoids: There are no available second-generation topical formulations of retinoids.
c. Third-Generation Retinoids

Topical adapalene 0.1% was approved for the treatment of acne in 1996 [101]. Adapalene binds selectively to RAR-β and RAR-γ, but it acts primarily via RAR-γ [101]. A meta-analysis of five clinical studies involving 900 patients with mild to moderate acne compared adapalene 0.1% gel and tretinoin 0.025% gel. After 12 weeks, both showed similar efficacy in reducing lesion counts, but adapalene had better local tolerability [110]. Topical adapalene reduces hyperkeratinization of pilosebaceous follicles and acne-related inflammation, with minimal side effects like redness and irritation [111].

Topical tazarotene gel (0.1%) was approved for treatment of mild to moderate acne in 1997 [101]. Like adapalene, tazarotene binds selectively to RAR-β and RAR-γ. Available in gel, cream, and foam, tazarotene offers greater potency but also potentially greater skin irritation [106,112,113]. A new lotion formulation (0.045%) aims to improve tolerability [106,112,113]. Often prescribed when tretinoin or adapalene are ineffective, tazarotene also has anti-inflammatory properties and targets hyperkeratinization and *C. acnes* proliferation [114,115]. It may be combined with benzoyl peroxide or antibiotics for enhanced inflammatory acne treatment, though skin irritation and redness are possible side effects [116].

Several studies comparing tazarotene, tretinoin, and adapalene for acne treatment have shown varied results. A trial comparing tazarotene 0.1% gel with tretinoin 0.025% gel found that tazarotene was more effective in reducing non-inflammatory lesion counts but caused more peeling and burning (*p* < 0.05) [117]. In a 12-week trial, tazarotene 0.1% gel had higher treatment success than adapalene 0.1% gel but also caused more peeling, burning, and erythema [118]. A retrospective analysis of seven studies revealed that tazarotene 0.1% gel had greater significant improvements than adapalene 0.1% gel and tretinoin 0.025% gel [119]. Lastly, a systematic review concluded that tretinoin works faster than tazarotene in reducing inflammatory lesions, while adapalene is better tolerated [104]. Despite these comparisons, factors like concentration and formulation affect tolerability and efficacy, with adapalene often being the most tolerated but least effective and tazarotene being the most effective but least tolerated.

d. Fourth-Generation Retinoids

Approved by the FDA in 2019 for acne treatment, trifarotene is the most recently approved topical retinoid [101]. Trifarotene is a fourth-generation topical retinoid with selectivity toward the RAR-γ located in the epidermis [103,104,120]. In the 12-week, double-blind, phase III PERFECT 1 and PERFECT 2 studies on 2420 patients with moderate facial and truncal acne, once-daily trifarotene 0.005% cream significantly improved success rates and reduced inflammatory and non-inflammatory lesions compared with the vehicle (*p* < 0.05) [121]. However, common adverse events, including application-site irritation (7.5% vs. 0.3%) and pruritus (2.4% vs. 0.8%), persisted [101]. Despite its potent, selective binding to the RAR-γ receptor, trifarotene did not fully eliminate the skin irritation often associated with retinoids. Although clinical trials demonstrated that trifarotene 0.005% cream significantly reduces lesions, comparative studies with currently available topical retinoids are yet to be conducted [106]. Theoretically, trifarotene’s RAR-γ selectivity might reduce adverse effects compared with existing topical retinoids; however, further studies are necessary to fully determine its optimal role in clinical practice.

Common side effects of topical retinoids include dryness, irritation, burning, and increased sun sensitivity [109]. To reduce these effects, patients should consider starting with a lower-strength formulation and gradually increasing it, applying the medication every other day or using short-contact therapy (removing the product after a short period) [122,123]. Additionally, the consistent use of moisturizers and sunscreen is essential. It is important to note that acne may initially flare up during the first few weeks of treatment [124]. While topical retinoids are the primary first-line treatment for both non-inflammatory and inflammatory acne, it can take over three months to see the full benefits of the therapy [3].

Topical retinoids are generally not recommended during pregnancy due to the potential risk of fetal malformations [124]. Better-studied options for pregnant patients include azelaic acid or topical clindamycin [124]. Tazarotene, in particular, should be avoided [124].

Research continues to focus on developing new formulations that improve tolerability and potentially increase effectiveness, such as tretinoin 0.05% lotion and tazarotene 0.045% lotion [106,112,113,125].

To maximize outcomes, topical retinoids are often combined with other acne treatments, including benzoyl peroxide and topical antibiotics to address the inflammatory component of acne, and hydrating agents (e.g., Altreno^®^) to combat the common side effect of dryness [109,126]. As shown in Table 2, tretinoin has been combined with clindamycin for the treatment of acne [102]. The tretinoin 0.1–benzoyl peroxide 3% cream (Twyneo^®^) received FDA approval as a once-daily application for moderate to severe acne vulgaris but has not yet been approved in the EU [127]. Also, adapalene has been formulated with benzoyl peroxide for treating acne. However, since both retinoids and benzoyl peroxide can cause skin irritation and dryness, combining them can intensify these effects. Gradual titration of such combination products may improve skin tolerance over time [102]. In 2023, FDA approved Cabtreo^TM^ (topical clindamycin phosphate 1.2%/adapalene 0.15%/benzoyl peroxide (BPO) 3.1%), the first triple-combination drug for acne [128]. Acne lesion reductions were significantly greater with clindamycin phosphate 1.2%/adapalene 0.15%/BPO 3.1% gel versus its dyads (BPO/adapalene; clindamycin/BPO; and clindamycin/adapalene) and vehicle gel by as early as week 4; however, it has not yet been approved in the EU. Topical 3% minocycline/0.3% adapalene foam is a combination in development for acne [129]. In the 12-week, double-blind, phase III PERFECT 1 and PERFECT 2 studies involving 2420 patients with moderate to severe acne, a combination of 3% minocycline and 0.3% adapalene significantly improved success rates, inflammatory lesion counts, and non-inflammatory lesion counts compared with the vehicle [129].

#### 3.1.2. Topical Benzoyl Peroxide

Topical benzoyl peroxide (BPO) is a widely used first-line antimicrobial treatment for mild to moderate acne [130]. It penetrates the skin and enters the pilosebaceous unit, where it generates free radicals that disrupt the cell walls of *C. acnes* bacteria [131]. BPO also exhibits mild comedolytic and anti-inflammatory properties and helps prevent the development of bacterial resistance to antibiotics—often offering superior results compared with topical antibiotics alone [132,133,134].

BPO is available as a gel, cream, or wash. Studies indicate that 2.5%, 5%, and 10% formulations are similarly effective, though higher concentrations increase the risk of irritant dermatitis [130]. Typically applied once daily, acne improvement may be seen within 5 days, with more noticeable results after 3 weeks and maximum lesion reduction after 8–12 weeks [130,135].

Common side effects include xerosis (dryness), scaling, erythema (redness), and hypersensitivity reactions [130,135]. BPO can also increase transepidermal water loss, affecting the skin’s barrier function [136]. Since continued use is needed for sustained effects, side effects typically lessen with reduced frequency or lower concentrations [135]. Importantly, patients should be aware that benzoyl peroxide can stain or bleach fabrics.

Combining BPO with other agents like topical retinoids or antibiotics enhances its efficacy in acne treatment [130]. Also, BPO helps reduce bacterial resistance when combined with antibiotics as a result of the bactericidal activity of BPO against antibiotic-resistant *C. acnes* [137]. For example, Benzamycin combines 3% erythromycin with 5% BPO in a topical gel, offering greater effectiveness and similar tolerability compared with BPO alone [138]. Also, the combination of clindamycin and benzoyl peroxide (BPO) has shown greater efficacy compared with using topical antibiotics alone, with 90% of patients using the combination reporting improvement at 12 weeks compared with 45% of those using clindamycin alone [132,133,134].

#### 3.1.3. Topical Antibiotics

Topical antibiotics are a key component in treating acne, offering both anti-inflammatory and antibacterial effects. They are particularly effective against inflammatory lesions but also reduce biofilm formation and subsequent microcomedones [139]. The American Academy of Dermatology (AAD) recommends topical antibiotics as a first-line treatment for mild acne and in combination with BPO or retinoids to combat antibiotic resistance [135].

Three topical antibiotics have FDA approval for acne treatment in both children and adults:

(a) Clindamycin: Available in various concentrations and formulations (lotions, foams, gels) [126]. To minimize resistance, clindamycin is frequently combined with BPO (BenzaClin, DUAC^®^) or tretinoin (Veltin) [126]. While generally well tolerated, dryness and skin irritation are possible side effects.
(b) Erythromycin: A possible alternative to clindamycin. However, concerns exist about higher rates of *C. acnes* resistance to topical erythromycin compared with clindamycin [126]. Therefore, it should be combined with other agents like BPO (Benzamycin^®^). Erythromycin is available as a monotherapy (gels, swabs, solutions). It is generally well tolerated, but can cause skin irritation [126].
(c) Minocycline: A tetracycline derivative. Topical minocycline 4% foam (Amzeeq™) demonstrates greater effectiveness than its vehicle alone [140,141]. This lipophilic formulation readily moves into the pilosebaceous unit [142]. While its precise mechanism is not fully understood, its potent antibacterial effects are well documented [142]. Studies indicate effectiveness in improving acne within 12 weeks, with continued improvement observed at 52 weeks [142,143]. Minocycline foam is generally well tolerated in trials, with the most frequent side effects being increased creatinine phosphokinase levels and headaches [140,141]. Topical minocycline 4% foam for acne has received FDA approval but has not yet been approved in the EU.

Bacterial resistance to both topical and oral antibiotics can develop within as little as 6 weeks of monotherapy [135,144]. Therefore, topical antibiotic monotherapy is generally discouraged. Combining with BPO minimizes resistance; BPO can be applied concurrently or as a wash prior to antibiotic application [133].

#### 3.1.4. Topical Azelaic Acid

Azelaic acid, a naturally occurring dicarboxylic acid found in barley and wheat, offers multiple benefits in acne treatment [145]. It functions as a comedolytic (preventing clogged pores), antimicrobial (fighting bacteria including *C. acnes*), anti-inflammatory (reducing acne-related inflammation), anti-keratolytic (regulating skin cell turnover), and antioxidant (protecting against cellular damage) [145,146,147]. Additionally, azelaic acid is effective in treating post-inflammatory hyperpigmentation [146,147].

Azelaic acid is available in both 15% gel (Finacea) and 20% cream (Azelex) formulations and is generally applied twice daily [147]. It is a first-line treatment option for mild to moderate inflammatory and non-inflammatory acne [147]. Azelaic acid is particularly well suited for those with sensitive skin or dyspigmentation concerns [147].

While generally well tolerated, some side effects can occur, including skin irritation, burning, redness, and itching [148]. Close monitoring is recommended in patients with darker complexions, as its lightening effect could be more pronounced [106]. Azelaic acid is considered safe for pregnancy (category B) and for lactating women [146].

#### 3.1.5. Topical Salicylic Acid

Salicylic acid, a beta-hydroxy compound, combats mild acne by acting as a comedolytic, keratolytic, and mild anti-inflammatory, while also possessing fungistatic and bacteriostatic properties [135,149,150].

Salicylic acid is widely available over the counter in concentrations ranging from 0.5% to 2% in both leave-on (gels, creams, lotions) and wash-off (cleansers) products [135]. It is a suitable option for patients who cannot tolerate stronger topical treatments like retinoids or BPO [135]. Salicylic acid can be combined with BPO for enhanced efficacy [146]. Additionally, higher concentrations are used in superficial chemical peels [146].

While generally safe, salicylic acid can sometimes cause skin irritation, dryness, itching, erythema (redness), and peeling [149,151]. In some cases, it might temporarily worsen inflammatory acne lesions [149].

A specific formulation, supramolecular salicylic acid, significantly improves acne and favorably modifies the skin microbiome to resemble that of individuals without acne [152].

#### 3.1.6. Topical Dapsone

Topical dapsone, a sulfone medication, offers antimicrobial, anti-inflammatory, and potentially immunomodulatory actions for treating acne [153,154]. It is available as a 5% or 7.5% gel and reduces both inflammatory and non-inflammatory acne lesions [155,156]. Topical dapsone has received FDA approval but has not yet been approved in the EU. It may be used for inflammatory papulopustular acne when initial therapies fail [146,157].

Topical dapsone is considered a second-line agent. However, for specific patient groups (women with inflammatory acne, those with darker skin tones, or patients with sensitive skin or contraindications to other treatments), it may be an appropriate initial therapy [158,159,160]. Due to its affordability, it could be particularly valuable in developing nations [155,156].

Studies have demonstrated its safety and efficacy, even in patients with glucose-6-phosphate dehydrogenase deficiency or sulfonamide allergies [160,161]. Therefore, unlike oral dapsone, glucose-6-phosphate dehydrogenase testing is not required for topical use [135]. Side effects are generally minimal but may include skin irritation [135].

Avoid combining topical dapsone with BPO, as this can cause temporary orange staining of the skin [135,146]. However, this discoloration can be washed off.

#### 3.1.7. Topical Sulfur and Sodium Sulfacetamide

Sulfur and sodium sulfacetamide are often used in combination to treat acne as their actions complement each other:

(a) Sulfur: Exhibits mild antibacterial and keratolytic properties, helping loosen dead skin cells to prevent clogged pores [162]. Sulfur interacts with the cysteine within skin cells to form hydrogen sulfide, disrupting disulfide bonds for its keratolytic effect [163]. Newer nanoparticle preparations may offer enhanced effectiveness against *Staphylococcus* bacteria, a contributor to acne pathogenesis [164,165]. Sulfur on its own can treat mild to moderate acne, but its results are improved when combined with sodium sulfacetamide or BPO [166,167,168,169,170].
(b) Sodium sulfacetamide: A bacteriostatic agent that disrupts bacterial DNA synthesis by inhibiting para-aminobenzoic acid [126]. Typically formulated as a topical lotion with 10% sodium sulfacetamide and 5% sulfur, it demonstrates significant acne lesion reduction (50–69% after 8 weeks; 78% after 12 weeks) [166,171].

Both sulfur and sodium sulfacetamide have generally well-tolerated side effects, which may include skin dryness, astringency (tightness), and itching [162,166,167]. Sulfur on its own has a noticeable odor, which is why it is often combined with sodium sulfacetamide for scent masking [168].

#### 3.1.8. Topical Clascoterone

Clascoterone (Winlevi^®^), approved by the FDA, is the first topical antiandrogen available for all sexes [172]. It is a groundbreaking topical acne treatment, the first with a novel mechanism of action since isotretinoin’s introduction in 1982 [173]. It received FDA approval in August 2020 but has not yet been approved in the EU. This topical steroidal antiandrogen offers both anti-inflammatory and antiandrogenic effects, making it a valuable tool for treating acne in both men and women [174,175].

Clascoterone functions by competitively inhibiting dihydrotestosterone (DHT) binding to androgen receptors (ARs) within the skin, particularly in sebaceous glands [173,174,176]. Androgen binding to the AR normally stimulates sebum production. By blocking this action, clascoterone decreases sebum production and reduces the production of pro-inflammatory cytokines [173]. Importantly, upon absorption, clascoterone is rapidly metabolized into an inactive form (cortexolone), minimizing the risk of systemic antiandrogenic effects [176].

Two large-scale, double-blind, vehicle-controlled phase III clinical trials (1440 patients with moderate to severe facial acne) demonstrated the superior efficacy of clascoterone 1% cream compared with the vehicle. Clascoterone significantly reduced both inflammatory and non-inflammatory acne lesions. Treatment success rates at week 12 were significantly higher with clascoterone (18.4–20.3%) compared with a placebo (6.5–9.0%). Also, a pilot study found clascoterone to be comparable to tretinoin 0.05% cream [176]. Importantly, clascoterone was well tolerated, with no systemic adverse effects reported. Local site reactions were primarily mild erythema (11.3–13.1%) and scaling/dryness (8.8–12.2%), occurring at similar rates to the vehicle control [174].

While clascoterone cream is rapidly metabolized, some adrenal dysregulation can still occur. Asymptomatic adrenal suppression was observed in about 7% of patients using high amounts of clascoterone in a phase II trial, which normalized after discontinuation [175]. Though typically used in women, systemic hormone modulation with oral contraceptives or spironolactone can be effective but have systemic side effects [175,176]. These options differ significantly from topical clascoterone.

Further comparative effectiveness studies are needed to fully establish its optimal role. However, it is likely to become a highly beneficial addition to treatment options for a wide range of acne patients. Importantly, this novel mechanism of action allows for combination therapies with other existing treatments.

**Table 1:** Topical treatments for acne.

Treatment Examples	Mechanism of Action	Indications	Common Side Effects	References
Retinoids (e.g., tretinoin, adapalene)	Normalizes follicular epithelial desquamation; anti-inflammatory	Mild to severe acne, depending on the formulation	Dryness, photosensitivity, initial acne flare-up	[3,103,104,105,106,107,108,109,111,112,113,114,115,116,120,122,123,124,125,126,177,178]
Benzoyl peroxide	Kills bacteria; peels out the inner lining of the hair follicle, causing skin peeling	Mild to moderate acne	Dryness, irritation, potential bleaching of clothes	[130,131,132,133,134,135,136,138,146,179,180,181,182,183]
Antibiotics (e.g., clindamycin)	Antimicrobial; reduces inflammation	Mild to moderate inflammatory acne	Skin irritation, resistance development	[126,135,139,140,141,142,143,144]
Azelaic acid	Kills bacteria; normalizes keratinization; anti-inflammatory	Mild to moderate acne	Pruritus, burning sensation	[106,145,146,147,148]
Salicylic acid	Helps break down blackheads and whiteheads; anti-inflammatory	Mild acne, comedonal acne	Skin irritation, dryness	[135,146,149,150,151,152]
Sulfur and sodium sulfacetamide	Antibacterial and keratolytic effects	Mild to moderate acne, rosacea	Dryness, skin irritation	[126,162,163,164,165,166,167,168,169,170,171]
Clascoterone (topical antiandrogen)	Blocks androgen receptors in the skin; reduces sebum production and inflammation	Moderate to severe acne	Local irritation, erythema	[173,174,175,176]

**Table 2:** Topical combinations for acne treatment.

Treatment	Formulation, Dose, Frequency	Common Side Effects	References
Tretinoin/Clindamycin	Gel 0.025%/1.2% daily	Xerosis, irritation, allergic contact dermatitis, erythema	[102]
Tretinoin/Benzoyl peroxide	Cream 0.1%/3% once daily	Xerosis, irritation, allergic contact dermatitis, erythema	[127]
Adapalene/Benzoyl peroxide	Gel 0.1%/2.5% once daily or 0.3%/2.5% once daily	Xerosis, irritation, allergic contact dermatitis, erythema	[102]
Benzoyl peroxide/Clindamycin	Gel 5%/1% once daily or 3.75%/1.2% once daily or 2.5%/1.2% once daily	Xerosis, irritation, allergic contact dermatitis, erythema, bleaching of fabrics	[132,133,134]
Benzoyl peroxide/Erythromycin	Gel 5%/3% once daily	Xerosis, irritation, allergic contact dermatitis, erythema, bleaching of fabrics	[138]
Clindamycin/Benzoyl peroxide/Adapalene (IDP-126)	Gel 1.2%/3.1%/0.15%	Xerosis, irritation, allergic contact dermatitis, erythema	[128]
Novel combined formulation in development: Minocycline/Adapalene (FCD105)	Foam 3%/0.3%	Xerosis, irritation, allergic contact dermatitis, erythema	[129]

### 3.2. Systemic Treatments

Systemic treatments for acne are utilized in moderate to severe cases, or when topical therapies are insufficient or not feasible. These treatments, which include oral antibiotics, hormonal therapies, and isotretinoin, target acne’s deeper pathophysiological mechanisms and offer broad-spectrum action against the factors contributing to acne development (Table 3).

#### 3.2.1. Oral Antibiotics

Oral antibiotics play a key role in managing moderate to severe inflammatory acne. They work by both reducing *C. acnes* bacteria and through anti-inflammatory actions [144,184]. Their efficacy extends even after discontinuation when used in conjunction with topical retinoids and/or BPO [185,186].

Tetracyclines remain a first-line treatment for acne due to their ability to inhibit bacterial protein synthesis. Among those, lymecycline and doxycycline are recommended as first-line oral antibiotics for the treatment of acne [187]. European guidelines limit the recommendation for systemic antibiotics to lymecycline and doxycycline and restrict the treatment period to three months to prevent the emergence of antibiotic resistance [188]. The US guidelines, Canadian guidelines, UK NICE guidelines, and European guidelines prioritize lymecycline and doxycycline over minocycline due to their comparable effectiveness and the higher incidence of severe adverse events associated with minocycline [188]. However, minocycline and doxycycline are widely used for their convenient once-daily dosing, compatibility with food, and effective penetration of follicles [189,190,191]. Sarecycline is a newer narrow-spectrum tetracycline approved by the FDA in 2018 [172]. However, it has not been approved by the European Medicines Agency (EMA). Sarecycline offers the potential benefits of specifically targeting *C. acnes* while minimizing disruption to the gut microbiome, potentially reducing the risk of antibiotic resistance [189,192,193,194]. Studies demonstrate sarecycline’s efficacy in treating moderate to severe acne [195].

Photosensitivity is a notable side effect particularly associated with doxycycline, highlighting the necessity for stringent sun protection and comprehensive patient education [196,197]. Moreover, to mitigate the risk of pill esophagitis, it is recommended that tetracyclines be consumed with sufficient water, and patients should refrain from lying down immediately post-ingestion [196,197]. Minocycline, while effective, is associated with a spectrum of potential risks including urticaria, vestibular effects, serum-sickness-like reactions, hyperpigmentation, drug reaction with eosinophilia and systemic symptoms (DRESS), and autoimmune hepatitis [198]. Sarecycline may have a lower incidence of these side effects. Also, doxycycline or lymecycline are often preferred over minocycline due to their comparably effective outcomes and lower incidence of severe side effects [135,199,200,201].

When tetracyclines are unsuitable (e.g., in patients under the age of 8, those with allergies, or other contraindications), other oral antibiotics can be considered. These include macrolides (erythromycin, azithromycin), penicillins (ampicillin, amoxicillin), trimethoprim/sulfamethoxazole, and cephalexin [135,202]. However, it is important to note that the evidence supporting their efficacy in acne treatment may be more limited.

Guidelines emphasize limiting oral antibiotic use to 3–6 months to curb antibiotic resistance, stressing the importance of continued topical treatment to maintain remission after discontinuation [187,199,201,202,203,204]. To minimize resistance, combination therapy with BPO is preferred over antibiotic monotherapy [205]. For adult women, hormonal therapies (spironolactone, combined oral contraceptives) may offer alternatives to antibiotics [195,206].

#### 3.2.2. Hormonal Therapies

Hormonal therapies play a crucial role in acne treatment, especially for women experiencing flares related to their menstrual cycle or those with underlying conditions such as polycystic ovary syndrome (PCOS). These therapies target androgen production and activity, as androgens (like testosterone and DHT) are major contributors to sebum production and acne development [207]. Hormonal therapies for acne include combined oral contraceptives (COCs) and antiandrogens.

COCs are a mainstay in hormonal treatment for acne, utilizing a combination of estrogen and progestin to suppress ovarian androgen production. They also increase levels of sex-hormone-binding globulin, which reduces the circulation of free testosterone, thereby diminishing sebum production [208,209,210]. FDA-approved COCs for acne include Beyaz (drospirenone/ethinyl estradiol/levomefolate), Estrostep Fe (norethindrone acetate and ethinyl estradiol), Ortho Tri-Cyclen (norgestimate and ethinyl estradiol), and Yaz (drospirenone and ethinyl estradiol) [207]. These options not only reduce the visible symptoms of acne but also regulate the menstrual cycle and reduce androgenicity. While improvement can take time, COCs are as effective as oral antibiotics over a longer course and are a first-line treatment for those with PCOS [146,207]. At 3 months, COCs are less effective than oral antibiotics, but by 6 months, they are equally effective in reducing acne [210]. However, their use must be carefully considered against potential risks, especially in patients prone to thromboembolic events [135,208]. Side effects include breast tenderness, mood changes, GI issues, weight gain, headaches, and breakthrough bleeding [208].

Co-cyprindiol (ethinylestradiol/cyproterone acetate) is used as a second-line treatment for severe acne in women, particularly when other treatments have failed [187]. However, it is advised against for long-term use due to safety concerns about the rare but cumulative dose-dependent increased risk of meningioma [187].

Spironolactone, an antiandrogenic diuretic, has proven effective against acne, particularly in female patients, by blocking androgen receptors to reduce sebum production [202,207,211]. Comparable in effectiveness to oral antibiotics, spironolactone is frequently prescribed for long-term management, often alongside oral contraceptives to enhance its antiandrogenic effects and to prevent pregnancy [212,213]. Recent studies suggest that the risk of hyperkalemia in healthy young women taking spironolactone is similar to the baseline risk in this demographic, indicating that routine monitoring of serum potassium may be unnecessary [214]. Common side effects include menstrual irregularities in women not on combined oral contraceptives and potential gynecomastia, though it is not linked to an increased risk of breast or gynecological cancers [213]. Given the risk of feminization of male fetuses, contraception is advised when prescribing spironolactone to women of childbearing potential [215]. Spironolactone can be initiated at doses ranging from 50 to 200 mg/day, adjusted based on clinical response and side effects, and is generally well tolerated [216].

Hormonal therapies are often preferable for long-term acne management compared with antibiotics [210]. Combining them with topical acne treatments can enhance their effectiveness. Careful patient selection is crucial, especially for those with hyperandrogenism or endocrine disorders, who may see the greatest benefit [207]. However, hormonal therapies may not be suitable for everyone due to contraindications like thromboembolic risk factors or specific medical conditions. For these patients, alternative treatments such as antibiotics, retinoids, or isotretinoin may be necessary. Interestingly, some studies suggest that the early initiation of hormonal antiandrogens could potentially decrease the need for prolonged courses of antibiotics in acne management [206].

It is important to be aware that progestin-only contraceptives and implants have the potential to worsen acne [217]. The composition of oral contraceptive pills matters: older generations (first and second) may exacerbate acne, while newer formulations (third and fourth generation) often have beneficial effects [218]. While hormonal treatments are not typically the first choice for acne in women, their use is becoming more widespread.

#### 3.2.3. Oral Retinoids

Oral retinoids are highly effective in treating acne by preventing keratin formation, which can block pores and contribute to acne development [202]. Typically reserved for severe cases that do not respond to topical treatments, well-known oral retinoids used for acne include isotretinoin, acitretin, and tretinoin [219].

Isotretinoin, a first-generation retinoic acid derivative, is indicated for severe recalcitrant nodular acne, but it is often used for resistant moderate to severe cases, acne causing scarring, or acne with a significant psychosocial impact [135]. It works by reducing sebum production, decreasing inflammation, normalizing follicular keratinization (reducing clogged pores), and reducing *C. acnes* abundance [184,220,221,222]. Isotretinoin has the potential to induce remission, with a cumulative dose of 120–150 mg/kg recommended [223]. Initiating therapy at a low dose may help minimize initial acne flares [204,224]. Some studies suggest continuing treatment for at least 2 months after full resolution to reduce the relapse risk [225,226].

Isotretinoin, known for its teratogenic effects, poses a risk of spontaneous abortion (20%) and embryopathy (18–28%) in exposed pregnancies [227]. Isotretinoin is absolutely contraindicated in pregnancy and lactation, requiring strict contraception during treatment and for one month afterward [68]. Common side effects like skin dryness (72.13%), cheilitis (94.25%), dry eyes (29.49%), and muscle aches (23.05%) are reversible and dose-dependent [228]. Muscle aches can sometimes be associated with elevated creatine phosphokinase (CPK) levels, indicating potential muscle damage [229,230,231,232]. While these elevations are often mild and temporary, monitoring CPK may be advisable for patients experiencing muscle-related symptoms or those engaged in frequent intense physical activity [229,230,231,232]. If significantly elevated CPK levels are detected, a reduction in isotretinoin dosage or a temporary cessation of treatment may be considered until CPK levels normalize [229,230,231,232]. Regular monitoring for liver and lipid abnormalities is recommended [233,234], with guidelines advising against full-blood-count tests [135]. A study among 13,772 acne patients showed frequent mild to moderate reversible lipid and liver enzyme changes, suggesting that baseline and periodic follow-up tests are sufficient [234,235,236]. Less common but significant potential side effects include skeletal effects (hyperostosis, premature epiphyseal closure), inflammatory bowel disease (IBD), and psychiatric effects (depression, emotional instability, anxiety, and, rarely, suicidal thoughts). However, the association with psychiatric effects remains debated, as acne itself carries a risk of depression and suicide [237]. Interestingly, a recent study suggests that isotretinoin use may be associated with a lower suicide rate compared with the general population [238]. Additionally, the association between isotretinoin and IBD remains controversial. Several studies and meta-analyses suggest no increased risk with isotretinoin, and antibiotic use may be a confounding factor in the reported association [239,240,241,242].

In cases where isotretinoin is ineffective or unsuitable for long-term maintenance, acitretin has shown success as a maintenance treatment [243]. Acitretin is a second-generation retinoid primarily indicated for psoriasis and keratinization disorders but offers potential in cases of nodulocystic acne that is resistant to isotretinoin [243]. Acitretin works by inhibiting epidermal growth and differentiation, providing a potential mechanism to control the formation of cysts and reduce inflammation [244]. While systemic retinoids are known for potential side effects like hypertriglyceridemia and hepatotoxicity, careful monitoring and dose adjustments can minimize these risks. Further research is needed to establish optimal dosing and patient selection criteria.

**Table 3:** Systemic treatments for acne.

Treatment Examples	Mechanism of Action	Indications	Common Side Effects	References
Oral antibiotics (e.g., tetracycline, doxycycline)	Antimicrobial, reduce inflammation	Moderate to severe acne	Gastrointestinal upset, photosensitivity	[135,144,184,185,186,187,189,190,191,192,193,194,195,196,197,198,199,200,201,202,203,204,205,206]
Hormonal therapies (e.g., oral contraceptives, spironolactone)	Regulate hormonal imbalance, reduce sebum production	Hormonally influenced acne, adult women	Mood changes, breast tenderness, thrombosis risk	[135,146,207,208,209,210,211,214,215,217,218]
Isotretinoin	Reduces sebaceous gland size and sebum production, anti-inflammatory	Severe scarring acne or acne not responsive to other treatments	Dryness, teratogenicity, elevated liver enzymes	[68,135,184,204,220,221,222,223,224,225,226,227,228,233,234,235,236,237,238,239,240,241,242]

### 3.3. Procedural Therapies

Emerging studies, although limited in scale, suggest that modalities such as laser and light devices, chemical peels, and intralesional steroid injections present viable options for mitigating acne symptoms and reducing scarring [135,245,246] (Table 4). The application of ablative and non-ablative laser technologies for acne scarring introduces options for significant resurfacing or collagen stimulation with less downtime, respectively, signifying that tailored approaches are required to optimize patient outcomes and minimize adverse effects [247].

Photodynamic therapy (PDT) stands out due to its strong evidential backing, which includes a meta-analysis of 13 randomized clinical trials encompassing 701 participants, highlighting its efficacy in reducing inflammatory lesion counts by a mean percentage of 15.97% [246]. PDT uniquely combines light energy with a photosensitizing agent, typically aminolevulinic acid or methyl aminolevulinic acid, to target pilosebaceous units and *C. acnes*, leveraging the bacterium’s innate production of photosensitizing porphyrins [246,248,249]. PDT has been posited to have a longer-lasting therapeutic effect than IPL [250,251].

LED (light-emitting diode) utilizing red and blue spectra have shown effectiveness in treating inflammatory acne [252,253,254,255]. IPL (intense pulsed light) treats inflammatory acne by killing *C. acnes*, reducing the size and number of sebaceous glands, and modulating inflammatory markers like TNF-α and TGF-β [250,254,256,257,258]. Pulsed-dye laser (PDL) therapy, alongside its role in inflammatory acne treatment, posits a longer-lasting therapeutic effect compared with IPL [250,251]. The use of the 1450 nm diode laser with a dynamic cooling device has shown moderate success in treating inflammatory acne and reducing excessive sebum production through various delivery methods [259,260,261]. Using stacked-pulse delivery appears to offer slightly better results, though individuals with darker skin (Fitzpatrick types IV–VI) should proceed with caution due to a higher risk of developing hyperpigmentation [259]. Another approach, a dual-regimen treatment that combines the low-fluence targeting of inflammatory lesions with a micropulsed moving mode applied across the entire face, has been found to be more effective than traditional high-fluency stamping. This method is not only less painful but also carries a reduced risk of causing hyperpigmentation [260]. The FDA-cleared AviClearTM (1726-nm laser) presents a targeted approach to treating mild to severe acne by selectively acting on sebaceous glands, with reported manufacturer outcomes suggesting sustained improvements post-treatment [262,263]. Similarly, the FDA has approved the 1726 nm Accure Laser System and TheraClear^®^, a combination of vacuum and broadband light technology, expanding the arsenal against acne [263].

The FDA’s recent approval of gold nanoparticles for acne treatment offers promising results through the targeted photothermolysis of sebaceous follicles [264,265,266]. Three weekly sessions combining these nanoparticles with diode laser pulses significantly improved inflammatory acne [267], while a similar protocol with a photopneumatic device effectively treated mixed acne [268]. A study also showed that the gold salt auranofin could inhibit the NLRP3 inflammasome in mice, suggesting anti-acne effects [269].

Chemical peels, ranging from superficial to medium depth, including agents such as glycolic acid, salicylic acid, azelaic acid, pyruvic acid, mandelic acid, phytic acid, and Jessner’s solution, have been scrutinized for their effectiveness in treating acne, post-inflammatory hyperpigmentation (PIH), and scarring. Despite the array of options, a robust analysis combining a meta-analysis with multiple split-face studies has yet to establish clear superiority among these treatments [270,271]. Superficial peels, particularly those using glycolic or salicylic acids, are noted for their efficacy in managing comedonal acne. They require a series of treatments for sustained results. A trial comparing these two acids revealed comparable outcomes in lesion reduction over 12 weeks, with the longevity of improvement notably extending up to 8 weeks after discontinuing salicylic acid treatment [271]. However, the application of chemical peels can lead to side effects such as erythema, dyspigmentation, and an increased infection risk. This highlights the necessity for a personalized treatment plan, considering factors like the patient’s skin sensitivity, healing ability, recent treatments, and overall health. Caution is particularly warranted for individuals with darker skin (Fitzpatrick skin types IV and higher) and those on oral retinoids due to a higher dyspigmentation risk [126]. For these patients, medium-depth peels are generally discouraged to avoid adverse pigmentary outcomes [263].

Non-ablative fractionated radiofrequency microneedling (FRFM) and combinations with isotretinoin have been effective in treating active acne and reducing scarring, with the adjunctive use of microneedling facilitating the enhanced delivery of topical treatments (retinoids and salicylic acid) and scar improvement [272,273,274,275]. Using isotretinoin in conjunction with other energy-based devices such as PDL and ablative fractional lasers has also been proven effective in managing active inflammatory acne [276].

Intralesional steroid injections provide an effective remedy for large inflammatory lesions and keloidal scars, albeit with caution due to potential local atrophy [277].

**Table 4:** Physical and laser therapies for acne.

Treatment Type	Mechanism of Action	Indications	Common Side Effects	References
Photodynamic therapy	Activates photosensitizing agents that kill bacteria and reduce sebum production	Severe acne, acne resistant to other treatments	Redness, swelling, skin sensitivity to sunlight	[246,248,249,250,251]
Blue-light therapy	Kills *C. acnes* bacteria using blue light without damaging the skin	Mild to moderate acne	Temporary redness and dryness	[252,253,254,255]
Chemical peel	Causes the top layer of skin to peel off, helps clear debris from pores	Mild to severe acne, depending on the peel’s strength	Redness, peeling, potential scarring	[126,263,270,271]
Intralesional steroid injection	Anti-inflammatory action and decrease in sebum production	Moderate to severe acne	Local atrophy	[277]

### 3.4. Diet Modifications 

The impact of diet on acne has been a subject of increased interest and research. Recent studies have begun to shed light on how certain dietary patterns may influence acne development, leading to the incorporation of diet modifications as complementary strategies in acne treatment plans (Table 5).

Previous studies have shown that dairy products and diets high in hyperglycemic carbohydrates boost insulin/IGF-1 signaling and lower insulin-like growth factor binding protein 3 (IGFBP-3) levels [278,279]. IGFBP-3, which binds to the retinoid X receptor-α, promotes cell growth and prevents cell death [280,281]. Meanwhile, IGF-1 reduces the presence of FoxO1 in the nucleus, triggering mTORC1. This activation of mTORC1 leads to an increase in keratinocyte and sebocyte proliferation and lipogenesis, contributing to acne development [282].

Also, adiponectin, known for its anti-inflammatory properties, is inversely associated with the glycemic load and dairy intake, suggesting that a diet with a high glycemic index can lower adiponectin levels, thus worsening acne severity [56]. Studies indicate that patients with acne have lower serum adiponectin levels compared with healthy controls, highlighting the potential of dietary management to modulate inflammation and acne severity [32].

Dietary patterns significantly impact gut diversity and the balance between beneficial and harmful bacteria, with a Western diet shown to decrease microbial diversity and skew the bacterial population in ways that may exacerbate acne. Studies reveal that acne patients exhibit distinct gut microbiota, characterized by decreased *Actinobacteria*, increased *Proteobacteria*, and a shift from *Firmicutes* to *Bacteroidetes*—an enterotype often associated with a Western diet [80,283]. This diet, rich in dairy, refined carbohydrates, and saturated fats, not only alters the gut microbiome but also triggers metabolic changes conducive to acne development [283]. Conversely, the adoption of a diet low in processed foods and high in vegetables, fruits, and omega-3 fatty acids can mitigate acne by promoting a healthier gut microbiota and reducing inflammation [284,285].

Acne patients often have lower levels of antioxidants like vitamin E, vitamin A, and selenium-dependent enzymes, contributing to acne-related inflammation [286]. Dietary fiber intake and plant-based antioxidants such as catechins in green tea and phytoalexins in red grapes have been shown to improve acne, possibly by reducing sebum production and inhibiting harmful bacteria [4,286]. A high-fiber diet beneficially alters the gut microbiome, improving insulin sensitivity and reducing hyperinsulinemia, thus potentially mitigating acne by decreasing IGF-1 levels, which are linked to increased sebum production and keratinocyte proliferation [4,82,287].

Vitamin D deficiency is more common among acne patients, suggesting a possible link between vitamin D levels and acne [288,289]. While some studies show that vitamin D can influence the expression of inflammatory biomarkers in sebocytes, suggesting a beneficial role in acne management, other research studies have found no significant association between vitamin D levels and acne development [290,291,292]. Additionally, vitamin D supplementation has been observed to positively alter the gut microbiome, potentially impacting skin health [293].

Zinc exhibits antibacterial properties against *C. acnes* and reduces inflammation [286]. Acne patients often have lower zinc levels, which correlates with the severity and type of acne [294]. Zinc can be used in pregnant women [69,295]. However, excessive iodine intake might exacerbate acne, indicating the need for balanced micronutrient intake for optimal skin health [286].

Biotin is a crucial water-soluble vitamin. Deficiency in biotin can occur as a side effect of isotretinoin treatment for acne, mainly due to its toxic effects on liver function, which reduce biotinidase enzyme activity [296]. This deficiency can lead to mucocutaneous and hair-related adverse effects [296]. A study has shown that supplementation with biotin (10 mg/day) alongside isotretinoin can alleviate these side effects [297]. The study found that biotin not only helps reduce the telogen (resting) hair ratio while increasing the anagen (growing) hair ratio but also assists in maintaining skin hydration [297]. These findings suggest that daily supplementation with 10 mg of biotin could be an effective strategy to mitigate the mucocutaneous adverse effects associated with isotretinoin treatment, making it a valuable addition to dietary modifications for patients undergoing isotretinoin therapy for acne.

**Table 5:** Dietary supplements for acne.

Supplement Examples	Mechanism of Action	Indications	Common Side Effects	References
Omega-3 fatty acids	Anti-inflammatory, modulates sebum production	Various acne types	Fishy aftertaste, gastrointestinal upset	[284,285]
Vitamin D	Modulates immune system, may reduce skin inflammation	Various acne types	Rare, potentially increased calcium levels	[288,289,290,291,292,293]
Zinc	Reduces inflammation and bacterial growth and modulates the immune system	Various acne types	Nausea, metallic taste	[69,286,294,295]

## 4. Emerging Therapies

Current research into acne treatment explores various facets, including sebaceous gland activity, inflammation, microbial flora, and even systemic influences like diet, unveiling promising candidates, each targeting different aspects of acne’s multifactorial nature (Table 6).

Hyaluronic acid (HA), a major glycosaminoglycan in the extracellular matrix, plays significant roles in skin cell functions. The HA-binding receptor, CD44, is expressed in human skin sebaceous glands. HA’s ability to downregulate lipid synthesis in a dose-dependent manner and significantly decrease sebum production positions it as a potential acne treatment [298].

Cannabidiol (CBD) is being researched for its potential to interact with the skin’s endocannabinoid system (ECS), which plays a crucial role in maintaining skin health and function [299]. CBD’s potential lies in its anti-inflammatory benefits, making it a candidate for treating inflammatory skin diseases [300]. Specifically, CBD exhibits sebostatic properties, effectively inhibiting the lipogenic actions of compounds like arachidonic acid and a combination of linoleic acid and testosterone. It also suppresses sebocyte proliferation through the activation of transient receptor potential vanilloid-4 (TRPV4) ion channels [301,302,303,304,305]. Given these properties, CBD is a promising candidate for the treatment of acne.

Biologic treatments have been explored in severe acne cases through small-scale studies. Agents targeting TNF-α, such as adalimumab and etanercept, have shown promise in treating aggressive forms of acne, including acne conglobata and fulminans [306,307]. Similarly, treatments inhibiting IL-17 and IL-23, namely secukinumab and risankizumab, respectively, have been effective in managing the stubborn SAPHO syndrome [308,309], underscoring the role of the IL-17/Th17 pathway in acne pathology [85]. However, a recent clinical trial revealed that CJM112, an IL-17A inhibitor with a unique target compared with secukinumab, did not outperform a placebo in reducing inflammatory acne [310]. Additionally, an RCT examining the efficacy of the anti-IL-1β inhibitor gevokizumab (XOMA 052) has been conducted, though results are yet to be published [106].

The gut microbiome has been increasingly implicated in the regulation of systemic inflammation and has an impact on various diseases. A study by Paetzold et al. did not utilize fecal microbiome transplantation but rather transferred healthy skin microbiome components to acne-prone individuals. This approach demonstrated that the recipient’s skin microbiome began to exhibit characteristics similar to that of the donor’s within a week, suggesting a potential for topical microbiome transplantation in dermatological treatments [311]. While this study opens new avenues for treating acne by altering the skin’s microbiome, it primarily focused on microbiome dynamics and engraftment without directly assessing clinical improvements in acne symptoms.

Building upon these insights, the theory suggests that enhancing the natural skin microbiota with topical probiotics could prevent the dominance of harmful *C. acnes* strains [312]. This approach is thought to work by promoting the production of antimicrobial substances from beneficial bacteria, reducing pro-inflammatory cytokines, and directly curbing the growth of *C. acnes* [312,313,314,315,316]. Research supports this, with studies showing that lotions containing enterocins from *Enterococcus faecalis* SL-5 improved inflammatory acne symptoms [317], and *Lactobacillus plantarum* effectively diminished acne lesion size and redness [318].

Oral probiotics have been found to elevate anti-inflammatory IL-10 levels in individuals with acne [319]. A study highlighted the efficacy of oral probiotics alone, in conjunction with oral minocycline, and as part of combination therapy, showing marked lesion reduction in all scenarios [320]. Furthermore, consuming lactoferrin-enriched fermented milk has been shown to reduce acne lesions [321], and a randomized controlled trial (RCT) demonstrated significant improvements in acne lesions and reduced *C. acnes* levels with a symbiotic supplement combining multiple probiotic strains and a botanical extract [322]. In a pioneering double-blind pilot study comparing the effects of a *Lactobacillus rhamnosus* GG supplement against a placebo over three months on 20 adults with acne, significant improvements were observed in the probiotic group [323]. Analysis of skin biopsies from these participants revealed a decrease in IGF-1 levels alongside an increase in FoxO1 gene expression, suggesting a positive regulatory effect on acne-related markers. These results suggest a promising role for probiotics as a beneficial and well-tolerated option for acne management, warranting further research to substantiate these preliminary findings.

Vaccines targeting *C. acnes* and its virulence factors, like CAMP factor 2, have shown promise in reducing acne-induced inflammation in mice studies [324]. However, currently, there is no clinical evidence for the effectiveness of a *C. acnes* vaccine in humans.

Bacteriophages, viruses that infect bacteria, offer an innovative approach to targeting acne-causing bacteria. Early research suggests their potential in reducing *C. acnes* and its associated inflammation, though clinical studies are needed to confirm their therapeutic benefits [325,326].

Designed antimicrobial peptides (dAMPs) represent a novel class of therapeutics, offering direct antimicrobial activity and immune modulation [327,328,329,330]. Their effectiveness against antibiotic-resistant strains of *C. acnes* in a preclinical model highlights their potential as a future acne treatment modality [331].

Phosphodiesterase (PDE) inhibitors have shown promise in treating acne through their anti-inflammatory properties. By inhibiting PDE, these compounds increase levels of cyclic adenosine monophosphate (cAMP), leading to reduced inflammation. Apremilast, a PDE-4 inhibitor, is one such drug that has demonstrated efficacy in reducing inflammatory lesions in acne, indicating the potential role of PDE inhibitors in acne treatment [332,333].

In light of the recognized role of the Akt/mTOR network in acne, there is an emerging interest in developing novel therapeutic agents targeting the mTORC1- and FoxO1-dependent signaling pathways to mitigate mTORC1/SREBP1-mediated sebum production, which plays a significant role in the pathology of acne [334]. One of the most widely used drug is isotretinoin, a powerful sebum suppressor that operates by increasing the nuclear expression of FoxO1, subsequently inhibiting mTORC1, which leads to the apoptotic death of sebocytes [335]. Beyond traditional drugs, natural dietary polyphenolic ingredients like epigallocatechin-3-gallate (EGCG) and resveratrol are potent mTORC1 inhibitors, effectively reducing sebaceous lipid production and ameliorating clinical acne manifestations [335]. A pilot study demonstrated significant clinical improvement in acne symptoms with the topical application of a resveratrol-containing gel [336].

**Table 6:** Emerging therapies in acne treatment.

Therapy Type	Mechanism of Action	Result	References
Hyaluronic acid	Acts as a major component of the extracellular matrix, downregulating lipid synthesis in a dose-dependent manner through its interaction with cellular receptors.	Significantly decreases sebum production, improving skin hydration and reducing oily skin appearance.	[298]
Cannabidiol	Interacts with the skin’s endocannabinoid system, helping to maintain skin health and function, while exerting anti-inflammatory and sebostatic properties.	Reduces inflammation and normalizes sebum production, leading to fewer acne outbreaks.	[299,300,301,302,303,304,305]
Biologic treatments (e.g., TNF-α inhibitors like adalimumab; IL-17 and IL-23 inhibitors like secukinumab)	Modulates the immune response by targeting and inhibiting specific cytokines involved in inflammation.	Reduces inflammation and the severity of acne symptoms, improving the overall skin condition.	[85,306,307,308,309,310]
Gut microbiome (microbial transplantation)	Transfers a healthy microbiome from a donor to an acne-prone recipient, fostering a microbial environment similar to that of individuals without acne.	The recipient’s skin microbiome adopts characteristics beneficial for preventing acne, reducing lesion formation.	[311]
Topical probiotics	Promotes the production of antimicrobial substances from beneficial bacteria and competes with pathogenic microbes on the skin.	Reduces pro-inflammatory cytokine levels and acne severity, promoting a healthier skin barrier.	[312,313,314,315,316,317,318]
Oral probiotics	Enhances the systemic immune function and modulates local inflammatory responses through increased levels of anti-inflammatory cytokines like IL-10.	Demonstrates a decrease in acne lesions and an overall improvement in skin clarity.	[319,320,321,322,323]
Vaccines	Targets *C. acnes* and its virulence factors, like CAMP factor 2, to reduce microbial-induced inflammation.	Leads to a significant reduction in acne-related inflammation and lesion count.	[324]
Bacteriophages	Specifically targets and kills *C. acnes* bacteria, thereby directly reducing the bacterial load and associated inflammation.	Early research suggests a potential reduction in acne severity and improved skin condition.	[325,326]
Designed antimicrobial peptides (dAMPs)	Provides targeted antimicrobial activity against resistant strains of *C. acnes*, while also modulating the immune response.	Reduces acne outbreaks and severity through effective bacterial control and reduced inflammation.	[327,328,329,330,331]
Phosphodiesterase (PDE) inhibitors	Increases the levels of cyclic adenosine monophosphate (cAMP), which leads to a decreased inflammatory response in the skin.	Shows promising reduction in inflammatory acne lesions, contributing to clearer skin.	[332,333]

## 5. Optimal Therapeutic Strategies for Varied Acne Severities

### 5.1. Acne Severity Grading

The accurate assessment of acne severity through standardized grading and classification systems is pivotal for guiding treatment strategies and evaluating therapeutic outcomes [337]. The Investigator Global Assessment (IGA) is the most widely utilized system in the United States due to its simplicity and proven correlation with patient self-assessments [338,339]. In assessing acne severity, the IGA Scale, sanctioned by the US FDA, serves as a critical tool, delineating five distinct grades. Grade 0 represents clear skin devoid of inflammatory or non-inflammatory lesions. Grade 1, described as “almost clear”, features rare non-inflammatory lesions alongside no more than one small inflammatory lesion. Grade 2, or “mild severity”, encompasses some non-inflammatory lesions with a few inflammatory lesions such as papules and pustules but excludes nodular lesions. Progressing to Grade 3, or “moderate severity”, the skin exhibits more non-inflammatory and some inflammatory lesions, with the inclusion of no more than one small nodular lesion. The highest severity, Grade 4, is categorized as “severe”, with numerous non-inflammatory and inflammatory lesions, including a few nodular lesions. Thus, the progression and escalating severity of acne is encapsulated and clinically assessed using this standardized scale [337,339,340].

### 5.2. Most Effective Treatment Combinations for Each Type

#### 5.2.1. Mild to Moderate Acne

For the treatment of mild to moderate acne, the combination of benzoyl peroxide and topical retinoids is highly effective, addressing key pathophysiological elements such as abnormal keratinization and microbial colonization while reducing sebum production [3,103,104,105,106,107,108,109,111,112,113,114,115,116,120,122,123,124,125,126,130,131,132,133,134,135,136,138,146,177,178,179,180,181,182,183]. Topical clindamycin in combination with benzoyl peroxide is recommended, leveraging the antimicrobial properties of clindamycin and the resistance-lowering effects of benzoyl peroxide, thereby enhancing therapeutic efficacy [126,135,139,140,141,142,143,144].

#### 5.2.2. Moderate to Severe Acne

In cases of moderate to severe inflammatory acne, a regimen comprising oral antibiotics, such as doxycycline or minocycline, alongside topical retinoids and benzoyl peroxide is advised to target deeper pathophysiological mechanisms, effectively reducing *C. acne* and mitigating antibiotic resistance [135,144,184,185,186,187,189,190,191,192,193,194,195,196,197,198,199,200,201,202,203,204,205,206]. Hormonal therapies, including oral contraceptives and spironolactone, are particularly effective for women exhibiting acne flares linked to hormonal variations, addressing androgenic contributions to acne pathogenesis [135,146,207,208,209,210,211,214,215,217,218].

#### 5.2.3. Severe, Nodular, or Treatment-Resistant Acne

Oral isotretinoin is recommended for severe, recalcitrant nodular acne or cases resistant to conventional treatments due to its profound impact on sebum production, follicular keratinization, and microbial populations, along with its anti-inflammatory properties [135,184,204,220,221,222,223,224,225,226,227,228,233,234,235,236,237,238,239,240,241,242]. In instances of severe inflammatory acne or acne fulminans where traditional treatments fail, systemic steroids or biologic agents such as TNF-α inhibitors may be utilized, providing significant anti-inflammatory effects and symptomatic relief [85,306,307,308,309,310].

## 6. Discussion and Conclusions

The complex relationship between the skin microbiome and acne pathophysiology has been highlighted, emphasizing the crucial roles of microbial diversity and specific bacterial species, such as *C. acnes* and coagulase-negative *Staphylococcus*, in this dermatological condition. These microorganisms are not merely present; they actively contribute to the development and exacerbation of acne through various mechanisms, including influencing sebum production and eliciting inflammatory responses.

The role of *C. acnes*, a key bacterium historically linked to acne, has been examined in detail. This bacterium thrives in oily environments provided by sebaceous glands and can trigger inflammation by activating the immune system. Its involvement varies, not just from person to person but also in its strain-specific behavior, which can either provoke or prevent inflammation. Recent studies suggest that specific strains of *C. acnes* are more commonly associated with healthy skin, while others are linked to chronic acne conditions, indicating the potential for targeted microbial treatments.

Probiotics and bacteriophages represent promising therapeutic avenues. Probiotics can help restore a healthy microbiome balance, potentially reducing acne severity by outcompeting harmful bacteria and reducing inflammation. Bacteriophages, which are viruses that infect bacteria, offer a novel approach to diminishing *C. acnes* populations without affecting the broader microbiome. This precision could prevent the development of antibiotic resistance, a growing concern with traditional acne treatments.

Environmental factors like diet and pollution were also discussed for their impact on acne. High-glycemic diets and increased dairy intake have been linked to increased sebum production and exacerbated acne symptoms. Pollutants can alter the skin barrier and microbiome, further aggravating or even triggering acne outbreaks. These factors highlight the importance of considering external influences in both the development and management of acne.

The genetic components of acne emphasize that genetic predispositions can influence an individual’s response to external triggers and treatment outcomes. The interplay between genes and the environment, including how they modulate the skin’s immune response and microbial landscape, is critical for understanding individual acne cases and for tailoring treatments accordingly.

In conclusion, this section of the paper advocates a holistic approach to acne treatment, one that incorporates not only topical and systemic therapies but also considers the patient’s lifestyle, environmental exposures, and microbial interactions. It calls for continued research into the gut–skin axis and its implications for acne, suggesting that future therapies could greatly benefit from integrating dietary and lifestyle modifications to support overall skin health and reduce acne severity. The ultimate goal is to move toward more personalized, effective, and sustainable acne management strategies that address the root causes of acne rather than just its symptoms.
